# Integrating VNIR–SWIR Spectroscopy and Handheld XRF for Enhanced Mineralogical Characterization of Phosphate Mine Waste Rocks in Benguerir, Morocco: Implications for Sustainable Mine Reclamation

**DOI:** 10.3390/s26010002

**Published:** 2025-12-19

**Authors:** Abdelhak El Mansour, Ahmed Najih, Jamal-Eddine Ouzemou, Ahmed Laamrani, Abdellatif Elghali, Rachid Hakkou, Mostafa Benzaazoua

**Affiliations:** 1 Geology and Sustainable Mining Institute (GSMI), Mohammed VI Polytechnic University (UM6P), Ben Guerir 43150, Morocco; ahmed.najih@um6p.ma (A.N.); rachid.hakkou@um6p.ma (R.H.); mostafa.benzaazoua@um6p.ma (M.B.); 2 Center for Remote Sensing Applications (CRSA), Mohammed VI Polytechnic University (UM6P), Ben Guerir 43150, Morocco; jamal-eddine.ouzemou@um6p.ma (J.-E.O.); ahmed.laamrani@um6p.ma (A.L.); 3 Department of Geography, Environment & Geomatics, University of Guelph, Guelph, ON N1G 2W1, Canada; 4 Materials and Environmental Chemistry Laboratory (IMED-Lab), Faculty of Science and Technology Gueliz, Cadi Ayyad University, Avenue A. Elkhattabi, BP549, Marrakech 40000, Morocco

**Keywords:** VNIR-SWIR spectroscopy, mine waste characterization, phosphate valorization, mine reclamation, circular economy

## Abstract

Phosphate is a crucial non-renewable mineral resource, mainly utilized in producing fertilizers that support global agriculture. As phosphorus is an indispensable nutrient for plant growth, phosphate holds a key position in ensuring food security. While deposits are distributed worldwide, the largest reserves are concentrated in Morocco. The Benguerir phosphate mining in Morocco generates heterogeneous waste (i.e., including overburden, tailings, and phosphogypsum) that complicates management and valorization, which is the beneficial reuse or value recovery from waste materials (e.g., use in cover systems, buffering, or other engineered applications). Therefore, it is essential to characterize their mineralogical properties to evaluate their environmental impact and possibilities for reuse or site revegetation. To do so, we integrate VNIR–SWIR reflectance spectroscopy with HandHeld X-ray fluorescence (HHXRF) to characterize phosphate waste rock and assess its reuse potential. For this purpose, field samples (*n* = 104) were collected, and their spectral reflectance was measured using an ASD FieldSpec 4 spectroradiometer (350–2500 nm) under standardized laboratory conditions. Spectra were processed (Savitzky–Golay smoothing, convex-hull continuum removal) and matched to ECOSTRESS library references; across the dataset, library matching achieved mean RMSE = 0.15 ± 0.053 (median 0.145; 0.085–0.350), median SAM = 0.134 rad, median SID = 0.029, and mean R^2^ = 0.748 ± 0.170, with 84% of spectra yielding R^2^ > 0.70. In parallel, HHXRF major and trace elements were measured on all samples to corroborate spectral interpretations. Together, these analyses resolve carbonate–clay–phosphate assemblages (dolomite commonly dominant, with illite/smectite–kaolinite, quartz, and residual carbonate-fluorapatite varying across samples). Elemental ratios (e.g., Mg/Ca distinguishing dolomite from calcite; K/Al indicating illite) reinforce spectral trends, and phosphate indicators delineate localized enrichment (P_2_O_5_ up to 23.86 wt % in apatite-rich samples). Overall, the combined workflow is rapid, low-impact, and reproducible, yielding coherent mineralogical patterns that align across spectroscopic and geochemical lines of evidence and providing actionable inputs for selective screening, targeted material reuse, and more sustainable mine reclamation planning.

## 1. Introduction

Phosphorus, primarily sourced from phosphate rock, is crucial for global food security as a key component of agricultural fertilizers [1,2,3]. The rising global population and intensifying agriculture are driving the increased demand for phosphate fertilizers, placing significant pressure on finite high-grade reserves [1]. Morocco plays a critical role in the global phosphate supply chain, holding about 70% of the world’s known reserves (≈68% in 2025) that are predominantly in extensive sedimentary marine phosphorite deposits [4]. Consequently, large-scale mining operations, such as the Benguerir mine in the Gantour Basin, are vital. However, these operations generate substantial quantities of waste rock, often at high stripping ratios (e.g., ~3 tons of waste per ton of ore at Benguerir [5]), presenting considerable challenges for sustainable environmental management [6]. This waste rock originates mainly from the overburden, interburden, and processing of low-grade ore. It typically comprises complex, heterogeneous mixtures that reflect diverse source geologies, including residual phosphate minerals (predominantly carbonate fluorapatite), carbonates (e.g., dolomite, calcite), siliceous materials (e.g., quartz, chert), and various phyllosilicates (e.g., illite, smectites, kaolinite) [5,6,7]. Although phosphate mine waste generally lacks the high sulfide content associated with acid mine drainage in other mining sectors, it poses distinct environmental risks [6]. These include land instability, visual impacts, and the potential mobilization of naturally occurring trace elements such as cadmium, uranium, arsenic, and fluoride under specific weathering conditions [8,9,10,11].

The mineralogical composition of mine waste fundamentally governs its geochemical and physical behavior: carbonate abundance controls pH-buffering, clay and apatite contents regulate trace-element mobility, clay-rich matrices dominate water retention and flow, and the proportion and fabric of fines influence overall stability [6,12,13,14,15]. Furthermore, these materials represent a potential secondary resource. Residual apatite, although sub-economic for primary extraction, may be recoverable through reprocessing [16]. Carbonates and silicate minerals could find applications in construction (e.g., road materials, concrete aggregates), agriculture, or environmental technologies, aligning with circular economy principles [5,17,18,19]. Effective environmental risk management and resource potential assessment necessitate a detailed understanding of the mineralogical composition and spatial heterogeneity of waste [5,20]. Traditional analytical techniques, such as X-ray diffraction (XRD), inductively coupled plasma mass spectrometry (ICP-MS), and optical microscopy, provide accuracy but are limited by cost, time, and spatial coverage [21,22]. These constraints hinder the development of optimized site-specific reclamation and resource management strategies.

To address this gap, rapid analytical techniques such as reflectance spectroscopy and portable X-ray fluorescence offer powerful alternatives for large-scale characterizations. VNIR-SWIR reflectance spectroscopy (350–2500 nm) can detect common rock-forming minerals based on diagnostic spectral absorption features arising from molecular vibrations and electronic transitions [23,24,25]. Key minerals in phosphate waste, such as fluorapatite, carbonates, and clays, exhibit distinctive features in this spectral range [26,27,28]. Handheld X-ray fluorescence (HHXRF) analysis complements spectroscopy by providing rapid, in situ elemental data crucial for validating spectral interpretations, especially in complex mineral mixtures [29]. Multiple analytical methods can be used to characterize the composition of mineralogical samples to provide a more complete picture. For instance, X-ray fluorescence (XRF) can provide critical elemental context for limestone, as shown by [30]. This can be complemented by spectroscopic analysis, a primary method for determining the mineralogical composition of materials, as exemplified by the study of Vesta using visible and infrared spectroscopy [31]. Furthermore, handheld XRF devices offer a rapid and effective method for in situ chemical characterization in environments such as mining operations [32].

This study aimed to characterize the mineralogical diversity of phosphate waste rocks from the Benguerir mine by integrating VNIR-SWIR spectroscopy with handheld X-ray fluorescence (HHXRF) analysis. Our primary objectives were to identify the dominant mineral assemblages and their diagnostic spectral signatures and to estimate semi-quantitative mineral abundances using spectral unmixing techniques [30,33]. HHXRF-derived elemental compositions were employed to validate and refine the spectroscopic interpretations. Furthermore, this study investigated mineralogical heterogeneity across different samples to assess compositional variability. The findings are discussed in the context of developing a rapid, cost-effective characterization framework with practical implications for site-specific reclamation planning and sustainable resource management strategies in Morocco.

## 2. Materials and Methods

### 2.1. Study Area

**Figure 1:**
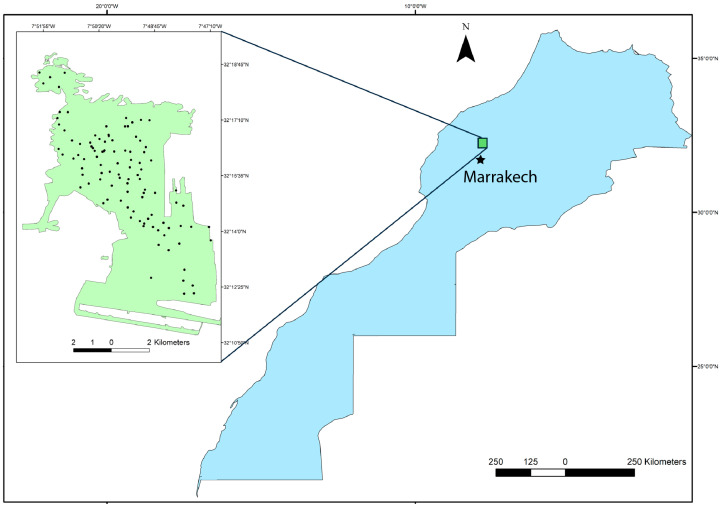
Map of the Benguerir phosphate mine in Morocco (green map), showing the locations of the 104 waste rock samples collected for this study. The map displays the distribution of the sampling points across the mine site (black dots).

The Benguerir open-pit phosphate mine (32°15′51″ N, 7°49′32″ W), operated by the OCP Group, is located approximately 70 km north of Marrakesh in the Gantour phosphate basin of central Morocco (Figure 1). The Gantour basin is an east–west elongated plateau roughly 120–125 km long and 20–30 km wide. The local phosphatic succession at Benguerir extends from the Maastrichtian to the Lutetian, consisting of phosphate layers interbedded with waste deposits, including numerous flint-bearing levels characteristic of the Gantour series [5]. Boujo’s monograph provides comprehensive mapping and facies characterization at the basin scale, identifying a western domain dominated by the Youssoufia facies and an eastern domain comprising the Benguerir, N’Zalet, and Tessaout Ouest/Est facies, with a transition zone near El Ouata [34]. Morocco holds the world’s largest phosphate reserves, approximately 50 Gt out of the global total of 74 Gt, highlighting the global importance of the Gantour deposits [4].

### 2.2. Data Collection and Sample Preparation

We have collected 104 waste rock samples from multiple accessible locations and depths (10–25 cm) across several large mine waste dumps (Figure 1). Sampling was designed to capture the full range of macroscopic variability in the waste rock materials, including differences in color, texture, degree of consolidation, and lithology. This strategy aims to ensure that the dataset is representative of the mineralogical and compositional heterogeneity of the waste deposits. The 104 samples were collected across the accessible extent of the study area to span (i) the range of depositional periods and pile types and (ii) the dominant lithological facies present in the waste rock piles. Some locations could not be sampled due to road inaccessibility and safety restrictions in abandoned zones.

**Figure 9:**
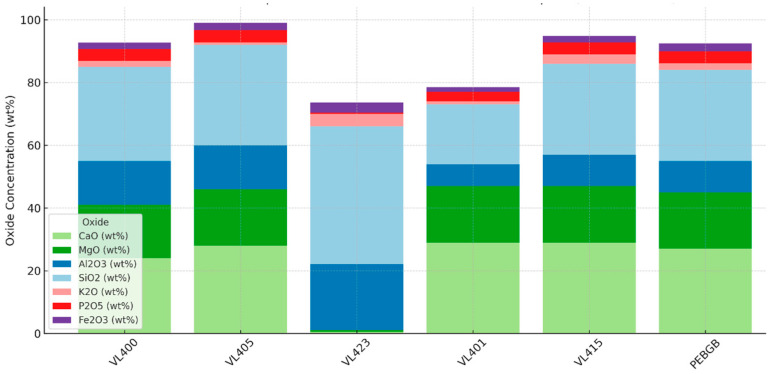
Major oxide composition (wt %) of representative Benguerir waste-rock samples measured by handheld XRF (HHXRF), illustrating variability among carbonate-, clay- and phosphate-bearing materials.

**Figure 10:**
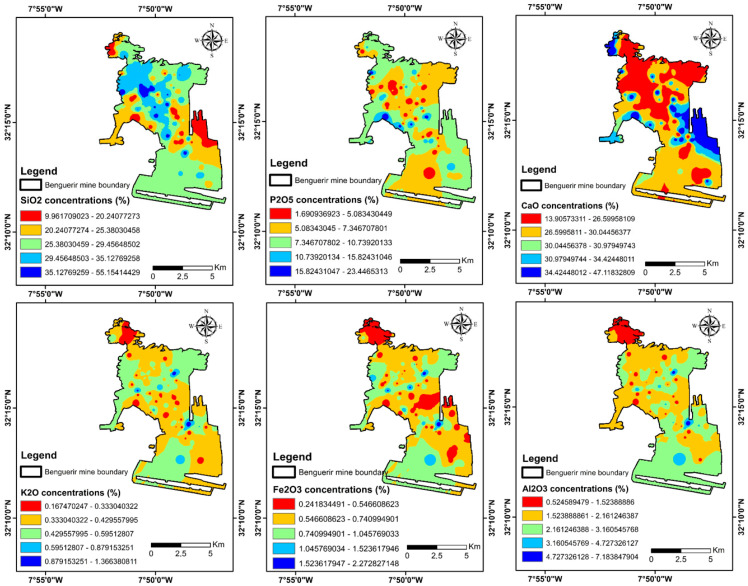
Spatial distribution of major oxides (%) from handheld XRF at 104 locations for different elements, SiO_2_, P_2_O_5_, CaO, K_2_O, Fe_2_O_3_, Al_2_O_3_; black outline shows the mine boundary (values in wt %).

To complement point measurements, we generated HHXRF-based spatial maps of major oxides (SiO_2_, P_2_O_5_, CaO, K_2_O, Fe_2_O_3_, Al_2_O_3_) together with a mineralogical dominance map; the resulting domains are reported in Section 3 (Figures 9 and 10).

To ensure consistency in the analytical results and minimize the influence of particle size on the spectral and XRF measurements, a standardized sample preparation protocol was implemented [19]. All collected samples were initially air-dried at ambient temperature and pressure. Representative subsamples were then crushed using a jaw crusher and subsequently pulverized with an agate vibratory ring mill to obtain a fine and homogeneous powder. The pulverized material was sieved to retain the fraction smaller than 2 mm, which was deemed optimal for both mineralogical and spectroscopic analyses. This uniform particle size fraction was used in all subsequent measurements to enhance data comparability and accuracy.

### 2.3. VNIR-SWIR Spectroscopic Analysis and Spectra Acquisition

**Figure 2:**
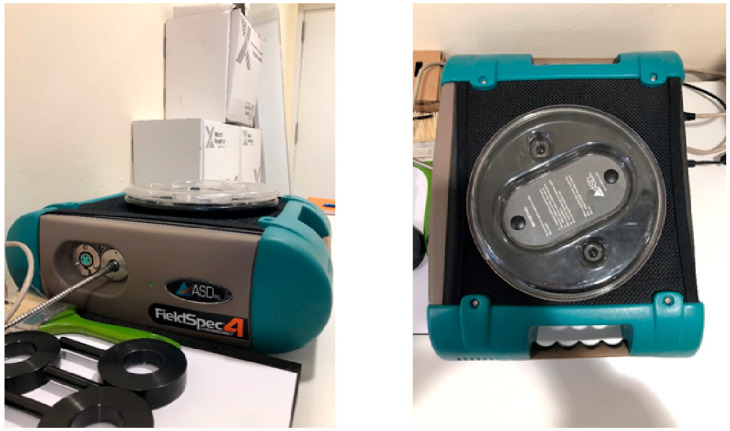
ASD FieldSpec 4 spectroradiometer used for data acquisition.

We collected the reflectance spectra using an ASD FieldSpec® 4 Hi-Res NG spectroradiometer (Analytical Spectral Devices Inc., Boulder, CO, USA), as illustrated in Figure 2. This instrument has a spectral resolution of ~3 nm in the VNIR (350–1000 nm) and ~8 nm in the SWIR (1000–2500 nm), with sampling intervals of 1.4 nm (VNIR) and 2 nm (SWIR).

All measurements were taken under controlled laboratory conditions using the instrument’s muglight probe. This setup ensured maximum illumination intensity and stable sample positioning, maintaining a fixed geometry to minimize variations caused by ambient lighting and viewing angles while optimizing the signal-to-noise ratio. Before each batch of measurements, the instrument was calibrated against a Spectralon panel (white reference) with a reflectance greater than 99% to ensure spectral accuracy.

The <2 mm powdered samples were placed into standardized sample holders and leveled to a consistent surface texture to reduce the variability in light scattering. Samples were jaw-crushed and pulverized in an agate ring mill to a fine, homogeneous powder, then sieved to <2 mm to remove coarse chips. Powders were packed and leveled in standardized holders and measured under a fixed Muglight geometry with Spectralon calibration and replicate acquisitions (plus an internal quartz standard) to minimize particle-size and surface-roughness effects. The same <2 mm powder was used for both VNIR–SWIR and HHXRF to ensure co-registered mineralogical and elemental measurements. While finer grinds (<150–200 µm) are common in some mineralogical protocols, we adopted this preparation to balance spectral fidelity with cross-technique comparability, consistent with established spectral theory on particle-size/scattering.

SWIR spectra were acquired with a Muglight accessory under fixed geometry. At the start of each session, we measured five internal standard reference replicates and computed the percent variation across replicates (relative to the replicate mean) across the whole spectrum. Sessions were accepted only if variation ≤ 6%; otherwise, measurements were halted and the system was re-stabilized (lamp warm-up ≥ 30 min, fresh white reference, inspection/cleaning of the Muglight window, and leveled, tamped packing in the Muglight cup), then re-tested until ≤6% was achieved. Only accepted sessions proceeded to soil measurements.

For each sample, five replicate spectra (each spectrum was the average of 30 individual measurements) were collected from slightly different positions on the sample surface. For accuracy and precision control, spectral measurements were harmonized using the Lucky Bay (LB) internal soil standard [35]. Replicates showing instrumental artifacts (e.g., saturation, negative values, or detector-join mismatch) were discarded. The remaining replicates were compared with the within-sample median across the full spectrum; scans that were clearly inconsistent (e.g., baseline shift or band-shape distortion on visual inspection) were treated as outliers and excluded. The accepted replicates were then median-averaged to obtain the representative reflectance spectrum for each sample [33,36].

Spectral data processing was performed using ViewSpec Pro (version 6.2) and custom Python 3.11.7 scripts. Raw reflectance spectra were clipped to 350–2500 nm and resampled at 1 nm spacing. A convex-hull continuum removal normalized each spectrum to its local continuum [33,37], and a Savitzky–Golay smoother (second-order polynomial, 7-point window) was applied to suppress high-frequency noise while preserving absorption features. Where noted, the first derivative was computed; second-order derivatives were not used. Continuum-removed (or derivative) spectra were matched to the ECOSTRESS library (splib07) using a weighted objective that emphasizes the phosphate window (2100–2300 nm; weight = 5). We assessed match quality with root-mean-square error (RMSE), spectral angle mapper (SAM), spectral information divergence (SID), and the coefficient of determination (R^2^). The top five candidates per sample were retained by the composite score (RMSE + SAM + SID) and visually inspected.

### 2.4. Chemical Analysis via HHXRF

Gazley and Fischer [38] and Hall et al. [39] measured the elemental concentrations using a handheld X-ray fluorescence analyzer (Niton XL5 from Thermofisher, Waltham, MA, USA). Measurements were performed directly on the prepared <2 mm sieved samples packed into standard XRF cups and covered with a thin (e.g., 4 µm) Prolene® X-ray film. The instrument operated using analysis conditions such as dual-beam analysis (e.g., 40–50 kV main beam, 10–15 kV low-Z beam) in an appropriate atmosphere (air) for a total time of 90–180 s per sample.

A user-developed calibration based on matrix types and certified reference materials (CRMs) was employed and regularly verified. This ensured data accuracy and allowed for the correction of potential matrix effects [39]. Concentrations of key major elements (expressed as oxides where appropriate, e.g., MgO, Al_2_O_3_, SiO_2_, P_2_O_5_, K_2_O, CaO, Fe_2_O_3_) and selected trace elements (e.g., Sr, U, As, Cd) were recorded.

To mitigate matrix effects and potential instrument drift, we verified readings after approximately every 20 samples, recorded any deviations, and adjusted the calibration if necessary. This consistent practice ensures that our elemental data remain accurate, particularly for major oxides such as CaO, MgO, and P_2_O_5_, which are critical for correlating mineralogical unmixing results with chemical composition.

### 2.5. Data Integration and Validation

A core component of this study was the integration of mineralogical data from VNIR-SWIR spectroscopy with elemental information obtained via HHXRF, enabling robust validation of mineral identification and abundance estimates. This cross-disciplinary approach ensured chemical consistency and enhanced the interpretability of the spectral signatures, particularly in complex mixtures.

Spectrally identified minerals were evaluated against elemental concentrations using established geochemical relationships. The following validation criteria were applied:

- Dolomite/Calcite: Samples exhibiting strong carbonate absorption near 2320–2350 nm are expected to show elevated CaO and/or MgO contents. The Mg/Ca ratio was a key discriminator between dolomite and calcite.
- The illite clay mineral content, identified by its diagnostic Al-OH absorption feature, was further confirmed by K_2_O (1.37 wt %), Al_2_O_3_ (7.25 wt %), and SiO_2_ (56.2 wt %). The K/Al ratios further supported the identification of K-bearing dioctahedral clays.
- Montmorillonite (Smectite Group): Samples interpreted as montmorillonitic showed high Al_2_O_3_ and SiO_2_, with variable levels of CaO or Na_2_O and consistently low K_2_O. These chemical patterns are consistent with those of non-K-bearing swelling clays.
- Kaolinite: Characterized spectrally by a distinct Al-OH doublet (~2165 and ~2200 nm), kaolinite was validated by high Al_2_O_3_ and SiO_2_ concentrations, coupled with low K_2_O, CaO, MgO, and Fe_2_O_3_. The Al/Si ratios were used to distinguish kaolinite from other aluminosilicates.
- Fluorapatite: The subtle absorption near 2150 nm, attributed to carbonate fluorapatite, was corroborated by the presence of P_2_O_5_ (up to 23.86 wt %), often accompanied by high CaO and elevated strontium (Sr), a typical apatite-associated element [40].
- Quartz: Although quartz lacks prominent spectral absorption features in the VNIR-SWIR range, its presence was inferred through its effect on the spectral continuum shape and was validated by the high SiO_2_ content in the absence of significant Al_2_O_3_ or other major cation oxides. This was particularly relevant for samples in which quartz was modeled as a minor endmember.

This integrative validation process enhanced the reliability of spectral library matching, helped resolve ambiguities between spectrally similar minerals (e.g., illite vs. smectites), and provided essential constraints for interpreting the results from spectral unmixing models. Unmixing was performed on a curated candidate set (quartz; calcite/dolomite; illite/kaolinite/montmorillonite/smectite; fluorapatite). For each spectrum, the top 4–5 library matches were fit using constrained least squares (non-negative; sum-to-one when stable). Post-fit residuals were inspected; cases with structured residuals near diagnostic bands were flagged as approximate and interpreted qualitatively.

## 3. Results

### 3.1. Spectral Signature Analysis

The VNIR–SWIR reflectance spectra collected from the 104 waste-rock samples exhibit a rich suite of absorption features consistent with mixtures of carbonate, clay, and phosphate minerals. Across the dataset, variation in band depth, shape, and precise position signals marked mineralogical heterogeneity, indicating that ostensibly similar samples differ in hydration state, crystal chemistry, and relative phase abundance.

First, nearly all spectra show pronounced H_2_O/OH overtones near ~1400 nm and combination bands around ~1900 nm. The variable depth and breadth of these features reflect differences in clay content, degree of hydration, and bonding environments in the phyllosilicate matrix. Building on this, a prominent Al–OH feature near ~2200 nm is ubiquitous but diagnostically diverse: sharp, symmetric bands centered at ~2200–2208 nm are consistent with illite or muscovite, whereas broader, slightly shifted absorptions point to smectite-group minerals. In several samples, a diagnostic doublet near ~2165 nm and ~2200 nm clearly indicates kaolinite, in agreement with reference spectra [27].

Moreover, strong absorptions within the ~2300–2350 nm interval highlight the carbonate contribution. A sharp minimum near ~2320 nm, often accompanied by shoulders or secondary minima between ~2335 and ~2350 nm, implicates dolomite as a dominant carbonate phase, with intensity variations tracking differences in carbonate abundance [26]. In addition, a subtle depression or inflection near ~2150 nm appears in many spectra; although low-contrast, this feature aligns with the expected response of carbonate-fluorapatite and supports the presence of apatite in complex mixtures [37,41].

Finally, several samples display broad visible-region effects, a general decrease in reflectance toward shorter (blue) wavelengths, and weak bands near ~500 nm and ~850–950 nm, consistent with minor iron oxides and hydroxides (e.g., goethite, hematite). While these Fe-related signatures can influence apparent color, they are not dominant in most cases.

**Figure 3:**
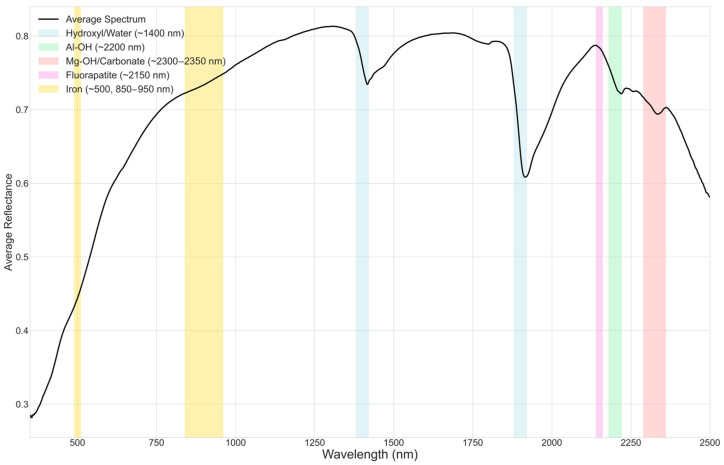
Averaged reflectance spectrum highlighting key mineral absorption features.

Taken together, the spectral evidence indicates that the majority of samples comprise intricate mixtures of carbonates and clays with minor phosphate and iron-bearing phases. This variability, illustrated in Figure 3, underscores both the heterogeneous nature of the waste material and the value of integrating spectroscopy with mineralogical and geochemical analyses for confident interpretation.

### 3.2. Mineral Identification and Semi-Quantitative Abundance Estimation

VNIR-SWIR reflectance spectra were analyzed using two complementary approaches: spectral library matching and linear spectral unmixing. This dual strategy enabled the robust identification of mineral phases and estimation of their relative abundances across 104 phosphate waste rock samples.

### 3.3. Spectral Library Matching

**Figure 4:**
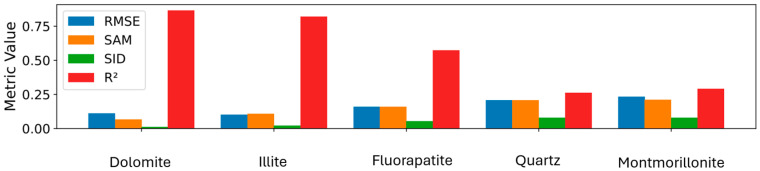
Matching metrics for the top five minerals of a random sample (VL401 sample).

Each sample spectrum was systematically compared with mineral reference spectra from the ECOSTRESS spectral library (splib07). Similarity was quantified using multiple metrics, including the Root Mean Square Error (RMSE), Spectral Angle Mapper (SAM), Spectral Information Divergence (SID), and coefficient of determination (R^2^). This combination of metrics provides a comprehensive assessment of the spectral fit, accounting for both amplitude and shape differences. (Figure 4)

A recurring set of minerals was identified throughout the dataset: dolomite, illite, montmorillonite, kaolinite, fluorapatite, and quartz. The combined use of multiple matching algorithms enhances the robustness of mineral identification, particularly in spectrally complex or compositionally mixed samples.

The first two minerals, dolomite and illite, in Figure 4, show strong spectral matches with R^2^ values ranging from approximately 60% to over 80% with low RMSE, SAM, and SID, indicating a high degree of similarity in both the shape and amplitude of their spectra compared to those of the sample. The fluorapatite, by contrast, shows a moderate global fit (R^2^ ≈ 60%) in this mixed carbonate–clay matrix. Because R^2^ can be depressed in mixtures, mineral identification does not rely on R^2^ alone; we consider RMSE, SAM, and SID together with visual inspection of the 2.10–2.30 μm phosphate feature. In the illustrated case, this feature is present and is therefore consistent with apatite in a mixed matrix, an interpretation supported by elevated HHXRF P_2_O_5_ with Ca and by XRD checks on representative samples. In contrast, the final two minerals, quartz and montmorillonite, displayed low R^2^ values and high RMSE, SAM, and SID metrics, indicating a poor spectral fit and minimal presence in the sample. This suggests that the spectral signature of the sample is predominantly composed of the first three minerals, with a clear dominance of dolomite and illite.

### 3.4. X-Ray Diffraction Validation

**Figure 5:**
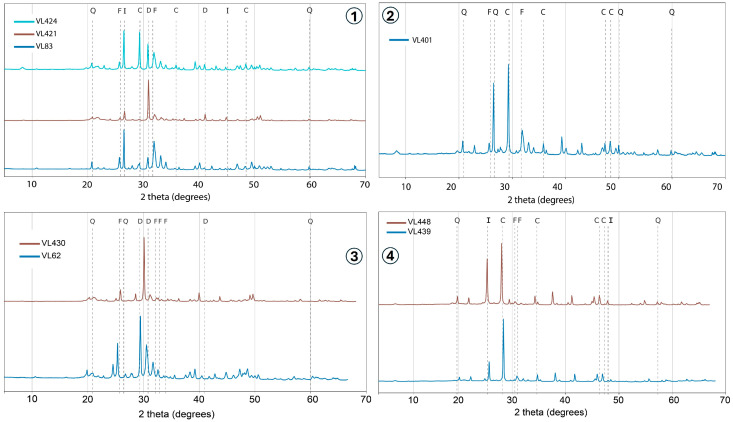
Powder X-ray diffraction patterns for eight representative waste-rock samples (1—clay-dominated group, 2—carbonate-rich group, 3—phosphate group, and 4—mixed group). Peak labels mark principal phases (Q = quartz, C = calcite, D = dolomite, I = illite, F = fluorapatite). Patterns corroborate the four groups identified from VNIR–SWIR and HHXRF. 2θ (Cu Kα) on the x-axis; intensities are arbitrary.

To corroborate the spectral identifications, we analyzed eight representative samples by powder X-ray diffraction spanning the four field classes (carbonate-rich, clay-dominated, phosphate-rich, and mixed). The patterns (Figure 5) confirm the phases inferred from VNIR–SWIR: calcite/dolomite in carbonate-rich samples, illite/kaolinite in clay-dominated samples, and fluorapatite in phosphate-rich samples. Minor deviations occur locally but do not alter the group assignments. This cross-check supports the qualitative interpretations drawn from the spectra together with the HHXRF chemistry.

**Figure 6:**
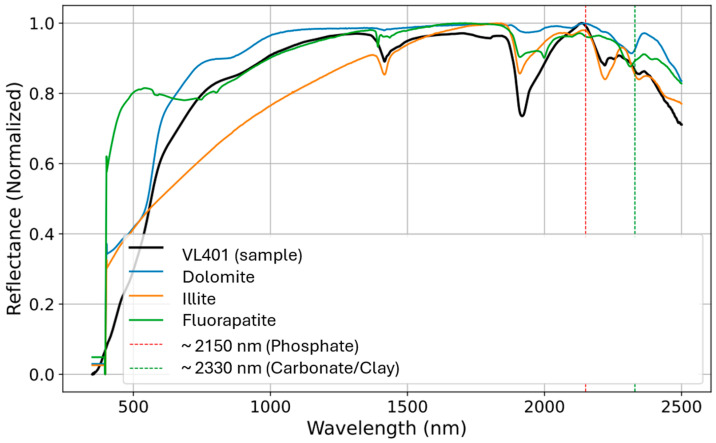
Matching metrics from the ECOSTRESS spectral library for the top three minerals of sample VL401.

Figure 6 provides a visual validation of the quantitative spectral-matching results for sample VL401. The plot shows the raw sample spectrum (black line) overlaid with the spectra of three top-matching minerals. The dolomite spectrum shows a good match, confirming its high R^2^ value. The illite spectrum also aligned well with the sample, particularly in the SWIR region. Although the fluorapatite spectrum shows some key similarities, there is a greater divergence from the sample line, consistent with its lower R^2^ value. The chart also includes dashed lines highlighting diagnostic absorption features at approximately 2150 nm (phosphate) and 2330 nm (carbonate/clay), which visually support the identification of these specific minerals.

**Figure 7:**
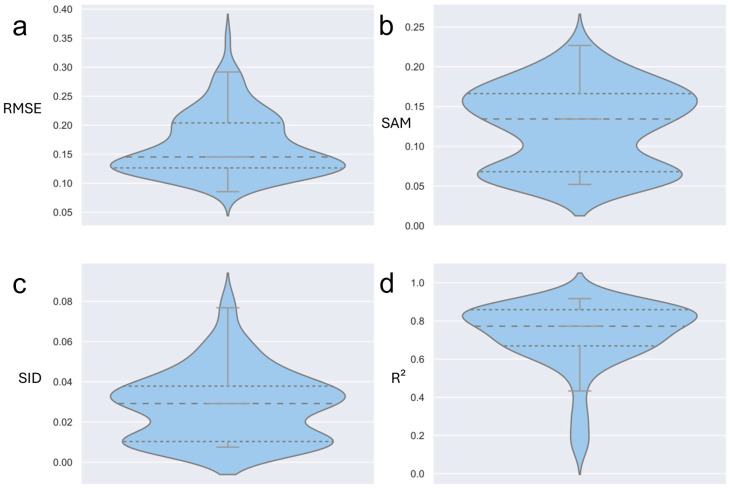
Violin plots (with embedded quartile boxes) showing the spread of (**a**) RMSE, (**b**) SAM, (**c**) SID, and (**d**) R^2^ (the max and min values are indicated within the plot) for all sample-library matches. Dashed lines indicate the medians, and dotted lines indicate the means.

While Figure 4 and Figure 6 illustrate a single representative sample (VL401), the same workflow was applied to all 104 spectra. Figure 7 summarizes the distributions of RMSE, SAM, SID, and R^2^. All four metrics display narrow interquartile ranges: the mean weighted RMSE is 0.166 ± 0.053, the median SAM is 0.134 rad (≈7.7°), and the median SID is 0.029. Importantly, 84% of spectra achieve R^2^ > 0.70. These cohort-level statistics verify that the curated library captures the dominant mineralogical variability of the waste-rock pile and that the matching procedure is robust. Summary values are provided in Table 1, and the full per-sample metrics are available in the Supplementary Materials.

### 3.5. Spectral Unmixing and Abundance Estimation

To move beyond qualitative identification, non-negative least squares (NNLS) linear spectral unmixing was employed to estimate the fractional abundance of each mineral phase in the reflectance spectra [42].

In this model, each observed reflectance spectrum is represented as a linear combination of known mineral endmember spectra:

R(λ) = Σ_i=1_^n^ a_i_ · E_i_(λ) + ε(λ)

where the following applies:

- R(λ) is the measured reflectance at wavelength λ,
- E_i_(λ) is the reference spectrum for mineral endmember i.
- a_i_ is the estimated abundance of mineral i,
- ε(λ) is the residual error between the modeled and observed spectrum,
- n is the number of selected endmembers.

The model was subjected to the following physical constraints: a_i_ ≥ 0 for all i.

This constraint ensured that all abundance values were physically meaningful. No sum-to-one constraint was applied to allow the presence of unmodeled phases.

The unmixing results revealed substantial mineralogical variability.

- Dolomite ranged from <10% to >85%
- Illite from trace to ~45%
- Montmorillonite up to 25%
- Kaolinite up to 40%
- Fluorapatite up to 20%
- Quartz generally <10%

**Figure 8:**
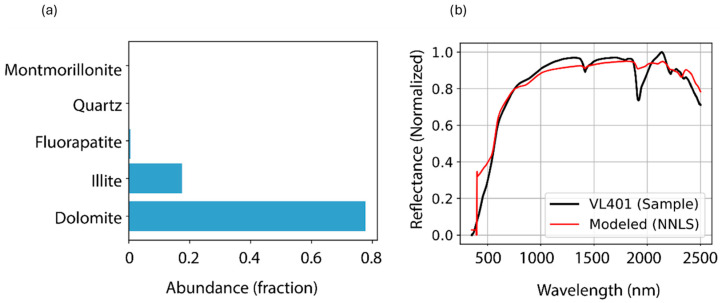
Spectral unmixing results for VL401: (**a**) relative abundance of each endmember identified, and (**b**) comparison of the measured reflectance curve (black) and the modeled reflectance curve (red).

The model quality was evaluated by comparing the reconstructed and measured spectra (Figure 8). Good agreement supported the reliability of the unmixing results, with minor residuals attributed to unmodeled phases, spectral overlap, or non-linear mixing effects [43].

### 3.6. HHXRF Elemental Composition and Validation

Handheld X-ray fluorescence (HHXRF) analysis provided quantitative elemental data that served as a critical validation tool for the mineralogical interpretations derived from VNIR-SWIR spectroscopy. Elemental concentrations across the 104 waste rock samples confirmed the presence of major rock-forming oxides and trace elements, which aligned well with the spectral variability observed (Supplementary Materials).

#### 3.6.1. Elemental Composition Trends

The HHXRF data revealed substantial compositional variability across the samples. The concentration ranges (expressed as oxides, based on common reporting conventions) were illustrated in Table 2.

These values are consistent with a mineral assemblage dominated by carbonates, aluminosilicate clays, minor phosphates, and accessory iron oxides (Figure 9).

#### 3.6.2. Cross-Validation with Spectral Results

Strong correlations were observed between the spectrally derived mineral abundances and measured elemental compositions. These correlations provided robust confirmation of the spectral interpretations and enhanced confidence in unmixing outputs.

- Carbonates (Dolomite/Calcite):
  Samples spectrally classified as dolomite-rich (>50%) consistently exhibited high combined CaO + MgO (≈35–45 wt %). The Mg/Ca ratios derived from HHXRF were characteristic of dolomite, distinguishing it from calcite-dominated materials (high CaO and low MgO).
- Illite:
  The spectral identification of illite was supported by K_2_O up to ~1.38 wt % in this dataset (median ≈ 0.40 wt %), together with high Al_2_O_3_ and SiO_2_. We therefore use the ~2200 nm Al–OH feature as the primary indicator, with K_2_O as a supportive proxy.
- Montmorillonite and kaolinite:
  Samples interpreted spectrally as smectite- or kaolinite-rich also showed high Al_2_O_3_ and SiO_2_ but crucially exhibited low K_2_O (<1.4 wt %). This elemental signature supports the differentiation of non-K-bearing clays from illite, even where spectral overlap occurred in the 2200 nm region.
- Fluorapatite:
  The residual apatite content, identified via the ~2150 nm absorption feature, was validated by the presence of P_2_O_5_. A positive correlation between estimated apatite abundance and P_2_O_5_ concentration was observed, with some samples containing up to 23.86 wt % P_2_O_5_. Elevated strontium (Sr), a common apatite substituent [40], was also detected in several samples, which further supports this interpretation.
- Quartz:
  Although quartz lacks distinct spectral absorption features, its presence was supported by high SiO_2_ concentrations in samples in which it was modeled as a minor component. Lowly associated Al_2_O_3_ and K_2_O levels helped differentiate quartz-rich spectra from those of aluminosilicates.
- Iron oxides:
  The Fe_2_O_3_ concentrations ranged from 0.06 to 2.3 wt %, corresponding to subtle absorptions in the visible spectrum (e.g., ~500 nm and ~900 nm) attributed to minor iron oxides such as goethite or hematite. These phases were not dominant but influenced the color and spectral characteristics of some samples.

This integrated mineralogical–chemical approach provides mutual validation across datasets. Spectroscopy offers rapid phase identification and abundance estimation, whereas HHXRF confirms compositional plausibility and refines interpretations, particularly for minerals with overlapping or weak spectral features.

### 3.7. Integrated Sample Interpretation

To demonstrate the practical value of integrating VNIR-SWIR spectroscopy with HHXRF elemental analysis, eleven representative phosphate waste rock samples were selected for a detailed cross-examination. These samples were chosen based on the clarity of their diagnostic spectral features, the robustness of their unmixing results, and the consistency of their elemental composition. The integrated dataset revealed a coherent and interpretable mineralogical framework, validating the combined methodology and highlighting its potential for rapid semi-quantitative assessment of complex geological materials.

#### 3.7.1. Carbonate-Rich Samples

Samples such as VL401, VL400, and VL02 illustrate the strength of this approach in identifying carbonate-dominated lithologies in the shallow subsurface. VL401, for example, exhibits a high spectral dolomite abundance (~78%), corroborated by a combined CaO + MgO concentration of 36.5 wt %, clearly reflecting a massive dolomitic matrix. Similarly, VL02 and VL400 showed spectral dolomite contents of >50% and ~85%, with CaO + MgO contents of 34.91 wt % and 36.25 wt %, respectively. The strong alignment between the spectral and elemental signals in these samples confirms the reliability of dolomite identification through the characteristic ~2320 nm absorption feature.

#### 3.7.2. Clay-Dominated Lithologies

The methodology also performed well in clay-rich samples, where the spectral unmixing effectively differentiated illite-bearing materials. Sample VL83 showed high illite spectral abundance (75%) and high K_2_O content (0.5 wt %), while VL421/24 displayed a comparable illite abundance (>60%) supported by K_2_O levels of 1.37 wt %. The clear presence of the ~2200 nm Al-OH absorption feature and the strong correlation with potassium concentrations demonstrate that VNIR-SWIR spectroscopy, when coupled with HHXRF, can confidently identify and validate K-bearing dioctahedral clays, even within complex mixtures.

#### 3.7.3. Phosphate-Rich Samples

Although the spectral identification of fluorapatite is inherently challenging due to the subtlety of its ~2150 nm feature, several samples nonetheless displayed consistent fluorapatite signals. VL62 and VL430 had spectral fluorapatite estimates of 50% each, which correlated well with high P_2_O_5_ concentrations (18.19 wt % and 15.72 wt %, respectively). VL421/24 exhibited a compelling case with approximately 40% fluorapatite spectrally and an outstanding 23.86 wt % P_2_O_5_ from HHXRF analysis, indicating potential as a localized phosphate enrichment zone.

#### 3.7.4. Mixed Assemblages and Balanced Mineralogy

Samples VL439, VL448, VL424, and VL18 show co-occurring dolomite, illite, and fluorapatite in the spectral unmixing (each appears among the top matches and in the endmember bars). Their HHXRF chemistry agrees with the spectral interpretation: VL439 (CaO + MgO ≈ 38.6 wt %, K_2_O 0.7 wt %, P_2_O_5_ 14.21 wt %), VL448 (≈31.5 wt %, 0.41 wt %, 7.0 wt %), VL424 (≈34.0 wt %, 0.35 wt %, 5.1 wt %), and VL18 (≈42.0 wt %, 0.37 wt %, 5.8 wt %). Taken together, these cases (with approximate spectral shares of dolomite ~40–55%, illite ~25–40%, and fluorapatite ~5–15%) demonstrate that the VNIR–SWIR + HHXRF workflow consistently captures multiphase mixtures and aligns mineral abundances with oxide concentrations across carbonate, clay, and phosphate groups.

The key strengths of the integrated interpretation are:

- High fidelity between mineral abundances predicted spectrally and oxide concentrations measured chemically.
- Multiphase capability: This approach is able to simultaneously identify and validate carbonates, clays, and phosphate minerals in complex waste matrices.
- Operational relevance: This integrated approach offers a scalable solution for rapid waste characterization in mining environments, supporting both environmental management and secondary resource recovery.

Overall, this detailed sample-level interpretation reinforces the credibility of the spectroscopy–HHXRF workflow. It not only supports mineral identification but also provides semi-quantitative estimates of compositional variability, enabling informed decisions for site-specific reclamation, resource valorization, and environmental monitoring.

### 3.8. Site-Scale Spatial Patterns

To place the point-based results in spatial context, we mapped the major oxides measured by HHXRF at 104 locations (Figure 10). Coherent domains and local hotspots are evident and agree with field observations as well as with the mineral groups verified above.

**Figure 11:**
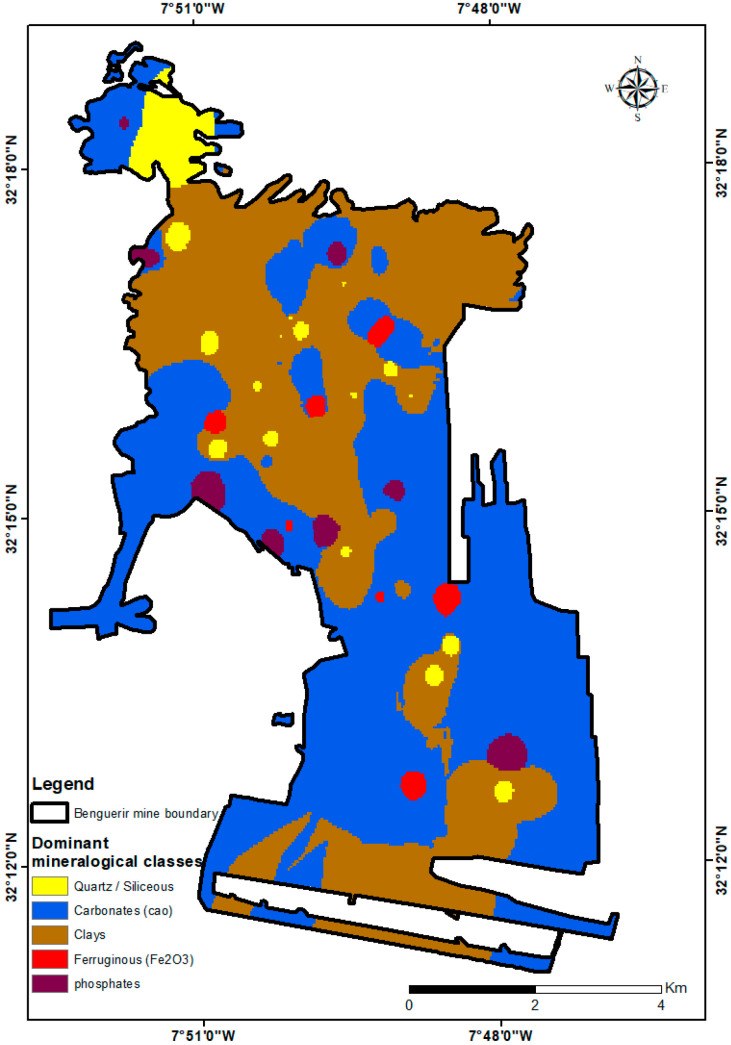
Mineralogical dominance across the study area derived from HHXRF composition: siliceous/quartz, carbonates, clays, ferruginous (Fe_2_O_3_-rich), and phosphates. Circles mark sampling locations; black outline shows the mine boundary.

Building on these trends, a mineralogical dominance map (Figure 11) summarizes the main lithological domains—carbonate-rich, clay-dominated, phosphate-rich, ferruginous, and siliceous—highlighting broad carbonate belts, clay/Fe zones, and localized phosphate areas across the Benguerir mine. Together, these maps synthesize geochemical and mineralogical variability at the site and naturally lead into the interpretation developed in Section 4.

## 4. Discussion

This study, through the synergistic application of VNIR-SWIR spectroscopy and HHXRF, provides clear mineralogical insight into the pronounced heterogeneity of phosphate mine waste at the Benguerir site. In addition, powder XRD on eight representative samples (Figure 5) qualitatively confirms the phase assignments inferred from the spectra—calcite/dolomite in carbonate-rich materials, illite/kaolinite in clay-dominated materials, and fluorapatite in phosphate-rich materials, thereby reinforcing the interpretation framework used here. This heterogeneity is not arbitrary but instead reflects the geological complexity of the Gantour Basin, where phosphatic strata are interbedded with marls, dolomitic limestones, cherts, and clay-rich horizons [7,44]. The excavation and dumping processes inherently mix these lithologies, creating a mosaic of mineralogical domains across the waste piles. 

The integrated workflow revealed significant variation in the relative abundances of dolomite, illite, and fluorapatite across samples. These compositional differences govern key chemical and physical properties, from pH buffering capacity (via carbonates) to water retention (via clays) and potential for residual phosphate recovery (via fluorapatite). Such findings underscore the need to move beyond generalized bulk characterizations and toward spatially resolved mineralogical mapping, particularly given the inherent heterogeneity confirmed in Moroccan phosphate waste contexts [6]. By enabling rapid, point-based measurements, the combined spectroscopy-HHXRF method offers a scalable solution for assessing heterogeneity at a resolution meaningful for operational and environmental decision-making.

The integrated method confirms the presence of residual carbonate fluorapatite throughout the waste rock matrix. Despite the often subtle spectral expression of apatite (~2150 nm), its detection was supported by corresponding P_2_O_5_ concentrations from HHXRF analysis, reaching up to 23.86 wt % in select samples. While average phosphate content remains below economic thresholds, the clear heterogeneity suggests that certain zones, particularly those with elevated spectral and chemical signals, could offer viable opportunities for secondary phosphate recovery.

Similarly, high-purity dolomite and other lithologies (e.g., flint, e) detected in numerous samples indicate potential valorization routes. Studies specifically focusing on Moroccan phosphate mine waste from Gantour and Benguerir have demonstrated its suitability for use in road construction and as aggregates in high-performance concrete, challenging previous classifications that limited its reuse [17,45]. This underscores the broader potential of phosphate waste not only as an environmental liability but also as a secondary resource that aligns with circular economy strategies [19]. Rapid screening using the integrated method facilitates the identification of such “resource hotspots” far more efficiently than conventional laboratory workflows, offering an actionable tool for strategic material reuse and value recovery. Armed with mineralogical and chemical results, operators can strategically allocate waste streams based on their properties. Carbonate-rich fractions (confirmed by elevated CaO + MgO and strong dolomite spectral signatures) might be reserved for creating buffering layers or neutralizing covers. Equally, identifying zones with elevated P_2_O_5_ could open possibilities for secondary phosphate recovery or serve as nutrient hotspots in ecological restoration protocols. Perhaps the most significant strength of this integrated approach lies in its practical utility for guiding sustainable reclamation strategies, particularly relevant given the environmental challenges of phosphate mining in Morocco’s semi-arid climate and the growing body of research focused on ecological restoration in these contexts [11,46,47]. By coupling rapid spectral data with elemental validation, mine operators can make informed decisions about material handling and environmental management in real time. For example:

- Informed material management: Carbonate-rich material (identified spectrally and confirmed by HHXRF) can be used to construct geochemically buffered landforms or encapsulate reactive waste, potentially utilizing concepts like store-and-release covers demonstrated effectively at other Moroccan mine sites [47]. Similarly, clay-rich units can be reserved for use as engineered covers or liners, reducing the need for imported materials [48].
- Optimized cover system design: Successful reclamation requires a clear understanding of underlying waste material properties. Mineralogical maps generated by this workflow can support the design of layered cover systems tailored to local hydrological and geotechnical conditions (e.g., sealing clay layers over permeable carbonate-rich zones), crucial for water management in arid regions [11].
- Targeted revegetation strategies: Plant establishment and growth are strongly influenced by substrate chemistry (pH, nutrients) and physical properties (water retention, texture). Mapping waste mineralogy will allow for the selection of appropriate plant species tolerant of the specific conditions (e.g., calcicole species in dolomite-rich areas) and guide targeted soil amendments (e.g., organic matter, specific nutrients based on deficiencies identified via HHXRF, and potential microbial inoculation [47]) only where necessary. Understanding the native flora naturally colonizing these disturbed sites, such as those documented at Benguerir and other Moroccan phosphate mines [49], is crucial for selecting appropriate species and enhancing revegetation success and ecological restoration outcomes on challenging substrates [11,49].

This study reinforces the methodological synergy between VNIR-SWIR spectroscopy and HHXRF. Spectroscopy offers rapid identification of mineral phases based on molecular vibrational features, particularly useful for detecting clays and carbonates. HHXRF, in turn, provides quantitative elemental data (e.g., for Ca, Mg, K, and P), which supports and constrains spectral interpretations, especially for spectrally ambiguous or weakly featured phases such as apatite.

However, like all methods, limitations exist. Both techniques are primarily surface-sensitive and require well-prepared samples to minimize heterogeneity, moisture effects, or surface contamination. Spectral unmixing accuracy depends on endmember quality, linear mixing assumptions, and the presence of secondary phases not well captured in the reference libraries [33,43,50]. The inherent non-linearity in light interaction within intimate mineral mixtures can introduce errors when using linear models [51,52].

Nevertheless, even with robust linear unmixing methods, intimate mineral mixtures may exhibit non-linear reflectance behavior that a pure linear model cannot fully capture. Approaches such as partially non-linear mixing models, support vector machines, or neural networks could refine the accuracy of abundance estimates by modeling subtle absorption overlaps and variations in grain size. Recent advances in unsupervised clustering and feature extraction for hyperspectral mineral data have demonstrated improved classification robustness and efficiency, as shown by [53] in their work on geological targets in the near-infrared. These methods complement supervised machine learning and non-linear unmixing models [51,52]. Furthermore, comprehensive reviews by [54] highlight the increasing adoption of machine learning frameworks for processing remote sensing data in mineral exploration, emphasizing their capability to enhance data quality, automate feature detection, and improve mineral classification accuracy. Incorporating these advanced algorithms could prove particularly useful in samples with weak or overlapping spectral features where linear models tend to show the largest residual errors. HHXRF data quality depends on proper calibration and correction for matrix effects [55].

Nonetheless, the integrated approach offers a practical, scalable, and cost-effective solution for waste rock characterization, dramatically reducing the time and labor required compared to traditional lab-based methods while providing mineralogical insights that are otherwise difficult to obtain in field contexts. From a practical standpoint, the portability of VNIR-SWIR spectrometers and handheld XRF drastically reduces both sample turnaround times and overall analysis costs compared to standard laboratory-based methods such as XRD or ICP. Although we have not conducted a formal cost analysis, preliminary observations suggest that the ability to rapidly scan large numbers of samples in situ could streamline decision-making in reclamation projects, enabling more frequent sampling and real-time adjustments to waste-handling strategies.

Building on this study, we propose an integrated roadmap to enhance the VNIR–SWIR–HHXRF workflow and broaden its impact on phosphate mine-waste characterization. To begin, systematic field-scale deployment and spatial mapping should extend the approach across entire waste dumps using grid-based sampling or transect profiling; when tightly coupled with GPS/GIS, the combined spectroscopy–HHXRF measurements can yield high-resolution mineralogical maps that delineate lithological boundaries, highlight localized phosphate enrichments, and flag zones suitable for reclamation or reprocessing. In parallel, three-dimensional characterization can emerge by fusing surface spectral data with subsurface information from drill cores, trenches, or geophysical surveys, thereby enabling volumetric models of waste composition that support selective handling, material zoning, and risk assessment. Moreover, enhanced spectral modeling and machine learning should address non-linear mixing and endmember variability through methods such as non-linear unmixing, support vector machines, and neural networks, alongside site-specific spectral libraries to improve endmember fidelity [19,51,52,56,57]. To translate mineralogical information into performance metrics, multivariate calibration (e.g., PLSR) can be developed to predict acid neutralization capacity (ANC), phosphate grade, and geotechnical parameters such as plasticity or abrasion resistance directly from spectral or XRF data, thereby strengthening operational decision-making [5,17]. In addition, waste valorization and recovery assessment should evaluate the feasibility of recovering secondary phosphate and carbonates by linking mineralogical mapping to liberation characteristics, beneficiation amenability, and trace-element behavior (e.g., Cd, U, Sr), with leaching tests or pilot beneficiation trials providing the critical bridge to process design [16]. Finally, because long-term stability and reactivity are dynamic, the same toolkit should be integrated into environmental monitoring: repeated surveys of reclaimed areas can track temporal shifts in surface mineralogy and geochemical signatures, offering early indicators of performance or failure risk and closing the loop between characterization, management, and monitoring [11].

Together, these directions represent a pathway toward building a more data-rich, adaptive, and sustainable phosphate mining framework, one in which mine waste is no longer a liability but a manageable, measurable, and potentially valuable resource. Looking ahead, broadening this workflow to incorporate comprehensive geostatistical mapping and non-linear spectral modeling stands to further refine our understanding of phosphate waste heterogeneity. By systematically quantifying uncertainties and optimizing handheld XRF calibrations, future studies can deliver an even more nuanced, reliable framework for both reclamation planning and potential secondary resource recovery. Therefore, this integrated approach can evolve into a precise, cost-effective blueprint for the sustainable management of phosphate mine wastes worldwide.

## 5. Conclusions

This study demonstrated that pairing VNIR–SWIR reflectance spectroscopy with handheld XRF provides a fast and practical workflow for understanding and managing phosphate mine waste at the Benguerir mine. This integrated approach delivers rapid, semi-quantitative mineralogical characterization of the complex waste rock. The synergy of joint spectral-elemental insight is key, as combined molecular and elemental evidence successfully resolves variations in carbonate, clay, and phosphate contents and confirms the presence of residual phosphate in certain areas. Furthermore, X-ray diffraction (XRD) analysis on representative samples provided qualitative support, validating the primary phase assignments made by the spectroscopy. The marked spatial and compositional heterogeneity of the waste is one of the significant findings of this study, which reflects the complex interbedded geology of the Gantour basin. Our analysis successfully identified the key mineral families driving this variability: carbonates (primarily dolomite and calcite), clays (such as illite and kaolinite), and residual phosphates (fluorapatite). Translating this to a site-scale view, the handheld XRF elemental maps and the derived mineralogical dominance map were crucial. These maps visually confirmed the heterogeneous structure, revealing coherent carbonate belts, distinct clay/Fe-rich zones, and localized phosphate spots across the mine site. The operational value of this portable workflow is its most critical outcome, as it enables real-time, data-driven decisions on-site. Mine operators can now more effectively guide reclamation planning, optimize cover system design by identifying suitable clay or carbonate layers, and strategically target hotspots for secondary phosphate recovery. This highlights significant valorization potential, as these carbonate- and clay-rich materials show promise for targeted reuse, aligning with Morocco’s circular-economy practices. Overall, the approach is field-ready for mine operators, geoscientists, and environmental engineers, providing a robust tool to enhance value, safety, and sustainability in phosphate-mine waste management.

## Figures and Tables

**Table 1:** Summary statistics for the four similarity metrics across 104 processed spectra. The full per-sample dataset is provided in the Supplementary Materials.

Metric	Mean	Median	Std-Dev	Min	Max
**RMSE**	0.15	0.145	0.053	0.085	0.350
**SAM (rad)**	0.137	0.134	0.050	0.052	0.227
**SID**	0.029	0.029	0.017	0.008	0.080
R^2^	0.748	0.773	0.170	0.130	0.917

**Table 2:** Major-oxide concentration ranges (wt %) measured by handheld XRF in Benguerir phosphate waste rock.

					Oxides		
	SiO_2_	Al_2_O_3_	CaO	MgO	K_2_O	P_2_O_5_	Fe_2_O_3_ (Total)
**Min (wt %)**	1.48	0.41	13.37	1.02	0.082	0.096	0.068
**Max (wt %)**	56.22	7.25	47.14	28.49	1.379	23.86	2.295

## Data Availability

Data will be made available on request.

## Supplementary Materials

The following supporting information can be downloaded at: https://www.mdpi.com/article/10.3390/s26010002/s1.

## Abbreviations

The following abbreviations are used in this manuscript:

ANC	Acid Neutralization Capacity
ASD	Analytical Spectral Devices
CRM	Certified Reference Material
ECOSTRESS (speclib07)	ECOsystem Spaceborne Thermal Radiometer Experiment on Space Station Spectral Library v07
GIS	Geographic Information System(s)
GPS	Global Positioning System
HHXRF	Handheld X-ray Fluorescence
ICP-MS	Inductively Coupled Plasma Mass Spectrometry
LB	Lucky Bay soil standard
NNLS	Non-Negative Least Squares
PLSR	Partial Least Squares Regression
RMSE	Root Mean Square Error
R^2^	Coefficient of determination
SAM	Spectral Angle Mapper
SID	Spectral Information Divergence
SRM	Standard Reference Material
SWIR	Short-Wave Infrared (1000–2500 nm)
VNIR	Visible to Near-Infrared (350–1000 nm)
wt %	Weight percent
XRD	X-ray Diffraction
XRF	X-ray Fluorescence
