# Supplementary material for: Integrating VNIR–SWIR Spectroscopy and Handheld XRF for Enhanced Mineralogical Characterization of Phosphate Mine Waste Rocks in Benguerir, Morocco: Implications for Sustainable Mine Reclamation
Source: Sensors (Basel). 2025 Dec 19;26(1):2. doi: 10.3390/s26010002 (PMC12787406; doi:10.3390/s26010002)
Supplement: Supplementary file 1 [file sensors-26-00002-s001.zip › Supplementary-Materials-Table-S1.pdf]

Supplemntary Materials:

Table S1: Detailed similarity metrics for each of the 104 processed spectra.

Samples	RMSE	SAM	SID	R^2	Score
**VL400**	0.2117469	0.16701794	0.03572132	0.62270362	0.41448615
**VL405**	0.21248103	0.18654559	0.05838997	0.32603233	0.45741658
**VL424**	0.16285227	0.14843549	0.03357946	0.75110154	0.34486721
**VL50**	0.17433458	0.14912933	0.03251445	0.72798625	0.35597837
**VL57**	0.16729777	0.15230867	0.03275102	0.70871939	0.35235746
**VL27**	0.15266404	0.14703555	0.03239874	0.7488543	0.33209833
**VL440**	0.22556668	0.17559121	0.04682415	0.6579153	0.44798204
**VL435**	0.14960767	0.14753585	0.03077285	0.73421322	0.32791636
**VL65**	0.16884013	0.14642452	0.03014104	0.72021897	0.34540568
**VL423**	0.34996157	0.21575082	0.06040311	0.43318264	0.62611551
**VL417**	0.21764236	0.16256272	0.03684175	0.67418481	0.41704682
**VL397**	0.13183732	0.13240726	0.02910587	0.80030856	0.29335045
**VL54**	0.21380924	0.10986409	0.01728055	0.69112817	0.34095388
**VL70**	0.28163425	0.18106273	0.04360478	0.59408709	0.50630176
**VL10**	0.20282369	0.16599844	0.0359038	0.62978871	0.40472594
**VL406**	0.15090984	0.10695294	0.02732501	0.79201135	0.28518779
**VL87**	0.17975398	0.15277764	0.03118021	0.69537274	0.36371182
**VL81**	0.21496536	0.16734119	0.04650226	0.68307815	0.42880881
**VL407**	0.19705029	0.17578436	0.0446088	0.64993758	0.41744345
**VL67**	0.17485679	0.1574842	0.03455338	0.69541048	0.36689437
**VL418**	0.2092937	0.1805922	0.05581372	0.27469443	0.44569963
**VL446**	0.21889743	0.17183547	0.04236474	0.6483266	0.43309764
**VL441**	0.19746903	0.16793192	0.03860059	0.66149213	0.40400154
**VL69**	0.16011853	0.14545568	0.03142969	0.7486224	0.3370039
**VL431**	0.24781395	0.17932311	0.04538359	0.61459136	0.47252064
**img2**	0.21625823	0.16897575	0.03780422	0.63523155	0.42303819
**VL113**	0.13743405	0.12464153	0.02870595	0.82173546	0.29078153
**VL437**	0.19057895	0.16147967	0.03522518	0.68809499	0.3872838
**VL396**	0.22911194	0.19095003	0.06142345	0.21811185	0.48148543
**VL438**	0.182373	0.16012559	0.03567732	0.68976324	0.3781759
**VL419**	0.19060656	0.17396139	0.04204013	0.64082011	0.40660808
**VL66**	0.28908572	0.1911024	0.04678897	0.52977896	0.52697709
**VL421**	0.18868847	0.15712783	0.03282512	0.66937699	0.37864142
**VL408-1**	0.09986699	0.1096671	0.02757699	0.86316307	0.23711108
**VL408**	0.14392478	0.13519631	0.02736782	0.75964395	0.30648891
**VL5**	0.15414517	0.14456732	0.03419308	0.75776284	0.33290557
**VL403**	0.14102876	0.13898129	0.02723447	0.743156	0.30724452
**VL433**	0.28972622	0.19185644	0.05031492	0.55761027	0.53189758
**VL79**	0.14503628	0.1372238	0.02929814	0.75721787	0.31155823
**VL15**	0.1276008	0.12864745	0.02850415	0.81183367	0.2847524
**VL35**	0.20941545	0.16168771	0.04010882	0.68348595	0.41121198
**VM4**	0.25746035	0.22688888	0.08044144	0.15889585	0.56479068
**img5**	0.10530057	0.11022016	0.0235464	0.85133912	0.23906712
**VL420**	0.24269902	0.20097403	0.05819186	0.56619298	0.5018649
**VL416-2**	0.17858045	0.16102023	0.03799913	0.69621066	0.37759981
**VL399**	0.1169763	0.12559624	0.02932337	0.81648515	0.27189591
**img4**	0.2106998	0.17384239	0.04442639	0.66951834	0.42896858
**VL432**	0.11220534	0.12563002	0.03082412	0.82691205	0.26865948

**VL429**	0.11260186	0.11979127	0.02529035	0.82877155	0.25768348
**VM1**	0.27144451	0.20418697	0.07122402	0.1296559	0.5468555
**point 215**	0.23825877	0.20495817	0.0768109	0.16807089	0.52002784
**VL425**	0.12839128	0.13137654	0.02861155	0.796745	0.28837937
**img3**	0.19887034	0.16076108	0.03429665	0.67246236	0.39392807
**VL444**	0.20728763	0.16805223	0.03651516	0.62030716	0.41185501
**VL41**	0.14390368	0.09050877	0.01383118	0.80349342	0.24824363
**VM6**	0.29164479	0.20784054	0.05671282	0.47729725	0.55619815
**VL436**	0.13714501	0.14493115	0.03530851	0.77023805	0.31738468
**VL442**	0.26157364	0.18676429	0.04804391	0.5798785	0.49638184
**VL4**	0.18890406	0.15643516	0.0341457	0.68189576	0.37948492
**VL13**	0.21618086	0.18304097	0.05433569	0.32362669	0.45355752
**VL85**	0.12906949	0.12626888	0.02731986	0.81184216	0.28265823
**VL73**	0.12380053	0.06415922	0.00955374	0.86114527	0.19751349
**VL416**	0.13850972	0.06369713	0.01031288	0.86912574	0.21251973
**VL402**	0.13904623	0.06806171	0.01080627	0.84577911	0.21791421
**VL426**	0.13324738	0.06981854	0.01014183	0.87561298	0.21320776
**VL428**	0.12918727	0.06080528	0.00960485	0.88197952	0.1995974
**VL401**	0.11207363	0.06683962	0.01142112	0.8652989	0.19033437
**VL422**	0.13641641	0.05989287	0.00929754	0.85399094	0.20560681
**VL411-2**	0.14566901	0.07937224	0.0121998	0.82628313	0.23724105
**VL112**	0.15674222	0.06127607	0.00878385	0.80396607	0.22680213
**VL413-2**	0.13029794	0.06172074	0.00811711	0.86075453	0.20013578
**VL415**	0.12837313	0.0672665	0.01117874	0.87080025	0.20681837
**VL14**	0.08543813	0.08818599	0.02444931	0.91690767	0.19807342
**VL434**	0.12298321	0.05621163	0.00752253	0.86676936	0.18671737
**VL447**	0.11081391	0.06119243	0.008223	0.8606782	0.18022934
**VL411**	0.12948699	0.05940676	0.00936202	0.82334837	0.19825577
**VL430**	0.12165808	0.05851647	0.0082956	0.87413895	0.18847015
**VL62**	0.12564179	0.07309164	0.01115717	0.86167039	0.20989061
**VL33**	0.13156019	0.06070403	0.00833134	0.8662477	0.20059556
**VL64**	0.12140826	0.05816848	0.00854711	0.86671276	0.18812384
**VL18**	0.11293065	0.06898846	0.00973343	0.86743104	0.19165254
**VL439**	0.13106573	0.06490534	0.00934394	0.82060029	0.20531501
**VL20**	0.11027682	0.11721936	0.01974063	0.80385916	0.24723681
**VL448**	0.120717	0.06467514	0.01401099	0.82026749	0.19940312
**VL443**	0.12184672	0.05848448	0.00808388	0.86034018	0.18841508
**VL25**	0.13443547	0.05617828	0.00888691	0.86420244	0.19950066
**VL9**	0.13789191	0.05200216	0.00758284	0.84141554	0.1974769
**VL414-2**	0.11929825	0.06802583	0.00990483	0.85874694	0.19722891
**VL404**	0.10214328	0.0732361	0.01077496	0.86464079	0.18615434
**VL58**	0.1227193	0.05780537	0.00786465	0.84130427	0.18838932
**VM3**	0.11881941	0.07303747	0.01031331	0.86937964	0.20217019
**VL409-2**	0.12662155	0.05781828	0.00845596	0.89171373	0.19289579
**VL427**	0.11239816	0.07118224	0.00985924	0.88117816	0.19343963
**VL7**	0.12030407	0.05633354	0.00794262	0.88154642	0.18458022
**VL53**	0.13243881	0.05690666	0.0079242	0.86072982	0.19726967
**VL83**	0.11823668	0.11901103	0.02073554	0.80792591	0.25798325
**VL410**	0.14549794	0.06564851	0.00944576	0.8149925	0.22059221
**VL408-2**	0.12228672	0.05492164	0.00757011	0.88395451	0.18477847
**VL17**	0.10846273	0.12119123	0.04428099	0.86942655	0.27393494
**VM5**	0.23059132	0.17184283	0.03828705	0.6142229	0.44072119
**VL33-2**	0.15852145	0.14325994	0.02800383	0.71392181	0.32978523

**VL409**	0.14239679	0.14150951	0.03271908	0.77529599	0.31662538
**VM2**	0.17601966	0.15888276	0.03601626	0.6900524	0.37091867
**VL410-2**	0.13378415	0.13352543	0.02780746	0.79060383	0.29511703
